# The Prediction Model of Medical Expenditure Appling Machine Learning Algorithm in CABG Patients

**DOI:** 10.3390/healthcare9060710

**Published:** 2021-06-10

**Authors:** Yen-Chun Huang, Shao-Jung Li, Mingchih Chen, Tian-Shyug Lee

**Affiliations:** 1 Graduate Institute of Business Administration, College of Management, Fu Jen Catholic University, New Taipei City 24205, Taiwan; hivicky92@gmail.com; 2 Artificial Intelligence Development Center, Fu Jen Catholic University, New Taipei City 242062, Taiwan; 3 Cardiovascular Research Center, Wan Fang Hospital, Taipei Medical University, Taipei City 116, Taiwan; leeshaojung@gmail.com; 4 Taipei Heart Institute, Taipei Medical University, New Taipei City 231, Taiwan; 5 Department of Surgery, School of Medicine, College of Medicine, Taipei Medical University, Taipei City 116, Taiwan; 6 Division of Cardiovascular Surgery, Department of Surgery, Wan Fang Hospital, Taipei Medical University, Taipei City 116, Taiwan

**Keywords:** National Health Insurance Research Database, NHIRD, CABG, machine learning, medical expenditure predict, feature selection

## Abstract

Most patients face expensive healthcare management after coronary artery bypass grafting (CABG) surgery, which brings a substantial financial burden to the government. The National Health Insurance Research Database (NHIRD) is a complete database containing over 99% of individuals’ medical information in Taiwan. Our research used the latest data that selected patients who accepted their first CABG surgery between January 2014 and December 2017 (*n* = 12,945) to predict which factors will affect medical expenses, and built the prediction model using different machine learning algorithms. After analysis, our result showed that the surgical expenditure (X4) and 1-year medical expenditure before the CABG operation (X14), and the number of hemodialysis (X15), were the key factors affecting the 1-year medical expenses of CABG patients after discharge. Furthermore, the XGBoost and SVR methods are both the best predictive models. Thus, our research suggests enhancing the healthcare management for patients with kidney-related diseases to avoid costly complications. We provide helpful information for medical management, which may decrease health insurance burdens in the future.

## 1. Introduction

Coronary artery bypass grafting (CABG) is the most common cardiac surgery to treat patients with severe coronary artery circulation blockages. After CABG, the patient will have the following two different situations: one is gradual recovery, the other is due to the complications that lead the patient to rehospitalization again [1]. Therefore, readmission is an essential outcome of CABG surgery, and it has a high incidence in 30 and 90 days [2,3,4]. Furthermore, it is a severe problem because it is directly related to the medical expenses that patients and hospitals must incur, substantially increasing healthcare costs and bringing a vast economic budget. However, the expenditure after CABG surgery remains poorly predicted. The various studies point out preoperative comorbidities, multiple complications, and medical expenses are essential variables that can affect the survival of CABG surgery patients [5,6,7].

This research used the National Health Insurance Research Database (NHIRD) to delineate this issue. It has been used widely and diversely in many academic studies [8]. Thus, the research results of NHIRD gradually become an indicator for clinical decisions, no matter in physicians or the government.

There are three aims in this research. First, we would use feature selection to identify the essential variables that affect postoperative expenditures. Secondly, we would use different feature selection methods to rank the essential variables. Last, we use different machine learning methods to build an appropriate medical expenditure prediction model for patients who underwent CABG. The information could effectively reduce medical expenditures, improve the quality of healthcare institutions, and provide essential references for medical management policy advice.

## 2. Materials and Methods

### 2.1. Data Source

Taiwan’s NHIRD has been built since 1995 and the coverage rate is about nearly 99%. NHIRD provides Taiwanese personal medical information, including primary demographic data and previous diseases. In addition, the NHIRD also covered all actual and most extensive healthcare data, including patients’ original outpatient, inpatient record, treatment, expenditure, diagnosis code, and admission dates. The codes were based on the International Classification of Disease, 9th Revision, Clinical Modification (ICD-9-CM); the 10th Revision was added to the database on 1 January 2016. This study was designed as a population-based study on 23 million national health insurance beneficiaries enrolled in Taiwan [9]. NHIRD provides a comprehensive long-term follow-up of all claimed records for the benefit of the NHI program. All personal information was anonymized and deidentified in NHIRD. Thus, Fu-Jen University’s ethics institutional review board in Taiwan was exempted from ethical review (C108121), and the requirement to obtain informed consent was waived.

### 2.2. Study Population

**Figure 1:**
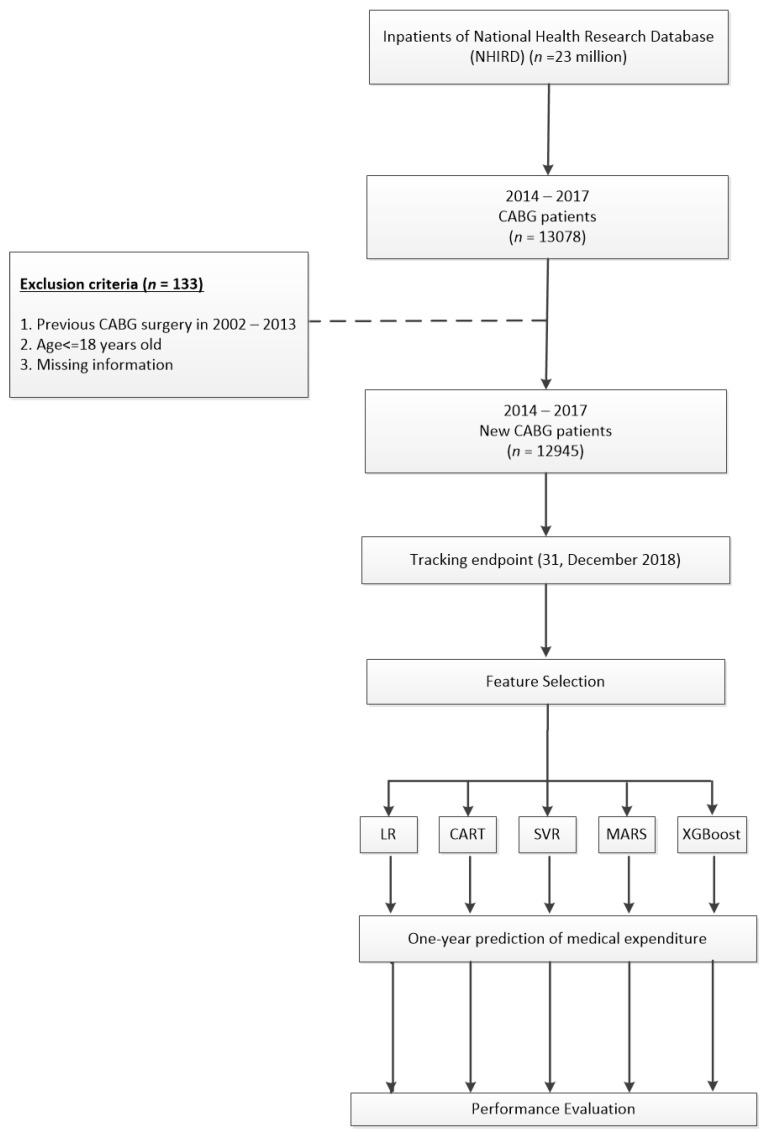
Flowchart of the patients who underwent the first CABG surgery between 2014 and 2017.

This research selected the patients who had accepted CABG surgery (procedure codes 68023A, 68023B, 68024A, 68024B, 68025A, 68025B) between 1 January 2014 and 31 December 2017, from the Taiwan NHIRD (*n* = 13,078). The date of newly CABG surgery is the index date. There were 133 patients that were not eligible for the study. To ensure that this study was only included the cases that received CABG operation for the first time, patients who had CABG surgery before the initial surgery year (*n* = 81) were excluded, and we also excluded the patients who were under 18 years old (*n* = 21) and missing information (*n* = 31) in this research. After excluding those unqualified patients for this study, 12,945 latest CABG surgery patients were included in our research from 1 January 2014 to 31 December 2017, and all followed up until 31 December 2018 (Figure 1).

### 2.3. Comorbidities and Risk Factors

The baseline characteristic variables in this study included sex (male/female), age, Charlson comorbidity index (CCI), and CHA2DS2-VAS scores [10,11]. Each patient’s comorbidities could be traced to the date before the CABG surgery (2002–2013). The comorbidities included diabetes mellitus (DM), hypertension, hyperlipidemia, myocardial infarction (MI), liver cirrhosis, congestive heart failure (CHF), coronary artery disease (CAD), peripheral vascular disease (PVD), acute pancreatitis, malignant dysrhythmia, atrial fibrillation (AF), transient ischemic attack (TIA), chronic kidney disease (CKD), acute coronary syndrome (ACS), chronic obstructive pulmonary disease (COPD), stroke, cancer, acute kidney failure (AKF), major bleeding, intracranial bleeding, end-stage renal disease (ESRD), and renal disease. Hospital reginal characteristics were as follows: hospital area type, hospital accreditation (medical center/non-medical center), and hospital ownership (public/private). The vessel numbers of percutaneous coronary intervention (PCI), hemodialysis, peritoneal dialysis, blood transfusion (94001C, 94002C, 4013C, 94015C, 94003C), and mechanical ventilation uses (57001B, 57002B, 57003B) in one year before surgery and during the corresponding surgery are also the risk factors in this study.

### 2.4. Variable and Outcome Definitions

This study used the total surgical expenditures of the corresponding CABG surgery and the patient’s medical expenditures in the previous year as predictive variables. The total surgical expenditures of each patient were calculated by the claimed records, including examination, anesthesia, treatment, drug, operation-related expenses, and other medical services during CABG hospitalization.

**Figure 2:**
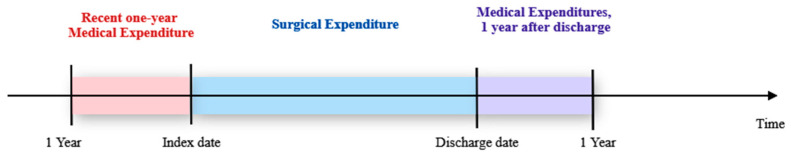
The definition of three different medical expenses associated with CABG surgery.

To define the primary outcome, this research used one-year cumulative expenditures after discharge to reflect the medical expenditures as primary outcomes. Therefore, we added up the total expense on outpatients and hospitalization after discharged for one year, and this variable is a prediction variable (Y) (Figure 2). All expenses are identified in New Taiwan dollars (NT$).

### 2.5. Feature Selection and Prediction Models Implementation

When doctors make clinical decisions, they must review the patient’s past medical records and current examination results one by one. This not only consumes time for searching, but also slows down the speed to make precise decisions immediately. Thus, feature selection (FS) is an essential preprocessing step before model prediction. By calculating different machine learning algorithms, removing irrelevant factors, we could reduce errors in clinical decisions and improve accuracy [12,13].

Medical expense is a continuous variable. Therefore, linear regression (LR) is often used for continuous numerical estimation, a model established by finding the relationship between the independent and dependent variables. In the training set, this research used five kinds of machine learning, including LR, classification and regression tree (CART), support vector regression (SVR), multi-variate adaptive regression splines (MARS), and XGBoost (extreme gradient boosting) to train by selecting the relevant features for medical expense prediction. In order to avoid overfitting, in the training process, we used five-fold cross-validation.

In more detail, we partitioned the training data into five stratified subsets, 80% of training data were used for training, and 20% of training data were used for validation. Subsequently, we repeated the above processes five times, each subset was used once as a validation dataset. After that, we obtained the average estimated results and used five different indicators to evaluate each prediction model.

#### 2.5.1. Linear Regression (LR)

Linear regression is the association between the dependent variable and one or more independent variables. Through the establishment of the regression model, the variable (y) can be predicted. Before building a prediction model, data must be a normally distributed.

#### 2.5.2. Classification and Regression Tree (CART)

CART can solve the regression and classification problem of multi-dimensional output. It is a kind of flow diagram tree structure; each node was the attribute variable. The branch is a test outcome, and the tree leaves present classification [14]. The method of CART for selection criteria is to use the Gini index. The Gini index is a measure of inequality, and it is usually used to measure income imbalance and can be used to measure any uneven distribution. A number between 0 and 1. 0 is entirely equal, and 1 is entirely unequal.

#### 2.5.3. Support Vector Regression (SVR)

The main algorithm of SVM is the “kernel”. When data cannot be linearly divided into lower dimensions, the kernel can transfer them to a higher dimensional divided linearly. SVR is an extension of SVM. In order to solve the problem of nonlinear, SVR is the model for considering the risk of structural, minimizing the generalization error, and maximizing hyper-plane margin to reduce the tolerated error [15,16].

#### 2.5.4. Multi-variate Adaptive Regression Splines (MARS)

Friedman proposed the MARS method in 1991 [17]. MARS is a non-parametric regression and flexible model, and it has consisted of the weighted sum of the basis splines piecewise polynomial functions. The optimal variable is hidden in the high-dimensional data. Through variable interactions, MARS can find the best variable easier [18].

#### 2.5.5. XGBoost (Extreme Gradient Boosting)

XGBoost methods were proposed by Chen et al. in 2016 [19]. It is an ensemble method based on decision tree methods. The framework in this method is gradient boosting, and model builds are sequential. Therefore, it can minimize errors, maximize models’ performance, and reduce tree construction time. The central idea in XGBoost is to make a new model to correct the errors in the previous training model, then make the prediction [20].

### 2.6. Validation Index

This study used different machine learning methods for the prediction of one-year medical expenses after discharge. The validation index of the model was the reference data for determining the quality and accuracy of the model, which depended on the model attributes.

In order to evaluate the performance of the model, this study used five different indicators to measure the prediction result, which was widely and easily understood. These five performance metrics represented the following three different types: absolute error, scaled error, and percentage error. The absolute error group contained the mean absolute error (MAE) and root mean squared error (RMSE), mean square error (MSE), mean absolute scaled error (MASE), and the group of percentage error includes mean absolute percentage error (MAPE) [21,22].

The mathematical formula of these statistical validation metrics for evaluating the models was demonstrated as follows in Table 1.

The indicators were frequently and widely used as a performance index among different prediction models [23]. The lower the deviation, the better the accuracy of the prediction model.

MAPE is one of the most popular indicators to use. If MAPE < 0.1, model has high accurate discrimination; 0.11 ≤ MAPE < 0.2, model has good discrimination; 0.21 ≤ MAPE < 0.50, model has acceptable discrimination; MAPE > 0.51, model is an inaccurate [23,24,25].

The above indicators were used to measure the prediction error in each model. Where *n* was the total amount of patients, *b* presented the actual medical expense, *a* represented the predicted medical expense.

### 2.7. Statistical Analysis

This research selected new CABG patients between 2014 and 2017, which was based on the disease’s demographic characteristics and history. All results were expressed as the number and percentages, *N* (%), for categorical variables. Means with standard deviation were presented as mean ± SD for continuous variables.

#### 2.7.1. Hardware Equipment

MOHW provides an environment for data analysis, the main analyzed computer CPU is intel i7-8700, the main host memory is 128 GB, the brand of system disk type is Western Digital (WD10EZEX) 1T.

Research data were provided from NHIRD, which is the largest volume of data in Taiwan. All analysis data will be stored in the other replacement hard disks (disk type: WD (DC HC310) 6T), which will be kept by the Health and Welfare Data Science Center (HWDC).

#### 2.7.2. Software

Patient data extraction was implemented in SAS version 9.4 (SAS Institute INC., Cary, NC, USA). Variable selection and model establishment is based on the relevant R statistical software (250 Northern Ave, Boston, MA 02210, R studio 3.6.1; https://www.rstudio.com/products/rstudio/). We used R package “stats”, “e1071”, “earth”, “rpart”, “XGBoost” to construct the prediction models LR, CART, SVR, MARS, and XGBoost, respectively.

## 3. Results

### 3.1. Demographic Characteristics of Study Population

A total of 12,945 new CABG surgery patients was selected from 1 January 2014 to 31 December 2017. The patient’s demographic characteristics and comorbidities are shown in Table 2. We analyzed 44 variables that possibly affected one-year medical expenses after discharge (Y). In the baseline factors, the patients’ age (X1) was 63.72 ± 10.65 years, the distribution in gender (X40) was 9,917 (76.61%) and 3,028 (23.39%) for males and females, respectively. CHA2DS (X2) was 3.29 ± 1.95 points, the score of CCI (X3) was 4.23 ± 2.82, and whether the patient had a significant illness (X41) was 16.28%. The factors during the CABG surgery (surgical variables) contained the following: surgical expenditure (X4) was 547,037 ± 436,611 (thousand NTD$), length of stay (X5) was 20.30 ± 12.02 days, blood transfusion (X6) was 7.94 ± 9.29 bags, mechanical ventilation use (X7) was 4.67 ± 15.55 days, the average of anastomosis was 2.40 ± 0.80 vessels (X8) and the average of PCI vessels (X9) was 1.19 ± 0.44.

The variables about one year before surgery were the average of hospitalization (X10), emergency department visits (X11; 1.27 ± 0.67), blood transfusion (X12; 4.08 ± 3.89 bags), mechanical ventilation (X13; 4.74 ± 11.44 days), medical expenditure (X14; 169,699 ± 247,396 thousand NTD$), hemodialysis (X15; 11.96 ± 5.01), peritoneal dialysis (X16; 10.65 ± 2.91), and 1.73 ± 1.13 PCI vessels (X17).

The comorbidities variables included the following: X18 diabetes mellitus (DM; 62.9%), X19 hypertension (49.21%), X20 hyperlipidemia (79.36%), X21 myocardial infarct (MI; 39.64%), X22 liver cirrhosis (2.84%), X23 congestive heart failure (CHF; 51.66%), X24 coronary artery disease (CAD; 93.06%), X25 peripheral vascular disease (PVD; 23%), X26 acute pancreatitis (3.34%), X27 malignant dysrhythmia (5.89%), X28 atrial fibrillation (10.55%), X29 transient ischemic attack (TIA; 31.97%), X30 chronic kidney disease (CKD; 29.45%), X31 acute coronary syndrome (ACS; 57.04%), X32 chronic obstructive pulmonary disease (COPD; 38.9%), X33 stroke (31.87%), X34 cancer (6.47%), X35 acute kidney failure (AKF; 11.7%), X36 major bleeding (23.32%), X37 intracranial bleeding (2.76%), X38 end-stage renal disease (ESRD; 6.41%) and X39 renal disease (28.82%).

X42 to X44 were hospital variables. The hospital area type (X42) was 15.13%, 62.10%, 20.54%, and 2.23% in central, northern, southern, and eastern, respectively. X43, different hospital ownership was 35.21% in public and 64.79 in private hospitals. Hospital accreditation (X44) was 61.89% in a medical center, and the non-medical center was 38.11%.

### 3.2. The Ranking Number of Feature Selection on CABG

After feature selection, we ranked the importance of each variable among different machine learning models that can provide helpful information for model building. Every algorithm has a different calculation. Thus, the variables selected were also different. For example, to determine the relative risk factors about the one-year medical expense after discharge, each important variable could provide helpful information through different feature selection methods. Huang et al. [5] point out that using fewer features was more efficient in model building.

This research used 44 variables [4,5,7,26,27,28,29,30,31], which depended on the physician’s clinical experience and literature review. Moreover, it used five different machine learning methods to predict after filtering factors, the highest score (10 points) was the most crucial factor, which will be the first on the rank; on the other hand, the lowest predictor was ranked the last (1 point). We listed the ranking degree and average in each variable in the following Table 3.

After screenings and analyses, the variable with a higher score was selected as the predicted value in this research. Through the calculation of different machine learning algorithms, each variable will have a different relative importance rank.

In the LR model, the most crucial variable was the surgical expenditure (X4). The other two variables, HD dialysis (X15) and medical expenditure (X14), were both from one year before surgery. Therefore, the top three essential variables of SVR, CART, and MARS are the same as LR. However, for XGBoost, the top two essential variables are still X4 and X14, and the third most important variable was CKD. Therefore, the essential variable in the LR, CART, SVR, MARS, and XGBoost models was surgical expenditure (X4; average point 9.8 points) and one-year medical expenditure before surgery (X14; average point: 9 points), and the number of HD (X15; average point: 8 points).

In general, we knew these three variables (X4, X14, X15) could affect one-year medical expenditures after discharge in CABG patients.

**Figure 3:**
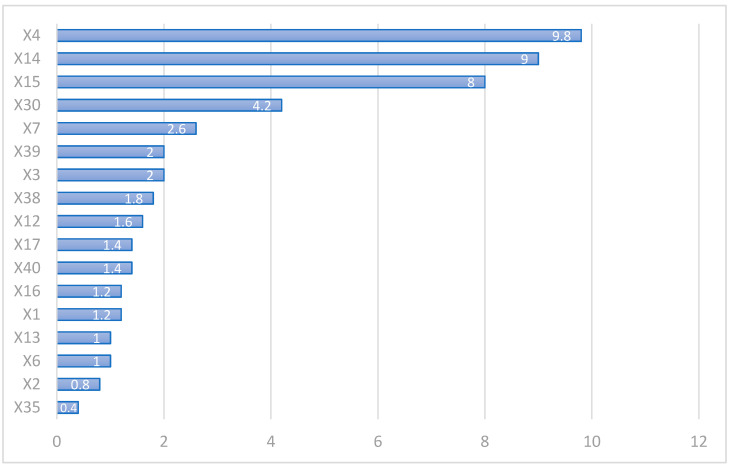
The average score after feature selection using five methods.

In order to clarify and simplify the predictors, we averaged the scores in each important variable for more equality, as shown in Figure 3. The result depicts the variables that possibly affect one-year medical expenditure after discharge.

The top five critical variables were surgical expenditure (X4), the one-year factors before surgery, medical expenditure (X14), the number of HD, CKD (X30), and the mechanical ventilation use during the CABG surgery (X7).

### 3.3. Performance of 5 Different Prediction Models

After the feature selection, we performed LR, CART, SVR, MARS, XGBoost prediction models. Then, to identify the lowest value in each indicator, we evaluated the following metrics: MAE, MSE, MASE, MAPE, and MAPE. For example, from the overall results in Table 4, after feature selection by CART and after XGBoost was used to make a prediction, MSE (0.0490) and RMSE (0.2214) were the lowest values.

We used the variables that were selected by MARS and SVR to build the prediction model. There were three indicators to show the lowest value, namely, MAE (0.1302), MASE (0.2067), and MAPE (0.0094). Thus, MARS only selected three variables and used SVR to make the best predictive model in this research compared to other combined methods.

## 4. Discussion

NHIRD provides a lot of medical information, and each patient could be traced for a long follow-up time. Therefore, we used NHIRD to make the medical expense prediction. The latest year of the NHIRD database is 2018. Therefore, we selected new CABG surgery patients between 2014 and 2017. The primary purpose of our study was to evaluate which factors could predict the one-year medical expenses after discharge of CABG patients, and build an expense prediction model. Most research discusses mortality, readmission, and the relationship between diseases and surgery [1,4,5,15,28,32,33,34]. However, only a few studies explored medical expenses, even forecasting. For example, Mehaffey et al. in 2018 [29], analyzed that each additional complication would cause an exponential cost increase. Baciewicz et al. in 2018 [28] referred that because sicker patients needed a high blood transfusion, it led to the increased expense. From the above results, we could know that the baseline variables, including age (X1), CHA2DS score (X2), CCI score (X3), CKD (X30), AKF (X35), ESRD (X38), renal disease (X39), major illness (X44), the variables one year before surgery (total medical expense (X14), blood transfusion (X12), mechanical ventilation use (X13), the number of HD (X15), PD (X16), and PCI vessels (X17)), the surgical variables (surgical expenditure (X4), blood transfusion (X6) and mechanical ventilation use (X7)), all positively influenced one-year medical expense after discharge.

In this study, we used multiple stages to analyze and predict the one-year medical expense after discharge. First, we used the feature selection method to find the essential variables that affect the medical expense. Secondly, after finding out the important variables, we selected five different machine learning models to build a prediction model and evaluate the performance. Besides, through feature selection, we found the folowing several exciting variables: CKD (X30), AKF (X35), ESRD (X38), and renal disease (X39). Although they are all associated with the renal condition, those variables do not have an exceptionally high ranking that is easy to be overlooked, they are topics worthy of further study. For example, Chou et al. [35] in 2014 evaluated that dialysis patients who underwent CABG surgery had better survival than PCI surgery; Chen et al. [36] analyzed that dialysis is associated with higher risk and mortality with CABG patients. Furthermore, Liao et al. [7] found that ESRD patients have a higher medical expense after CABG surgery. From the above results, it could be known that for kidney disease patients who accepted their first CABG surgery, a one-year expense after discharge would be relatively high.

The medical expenditure in preoperative one-year (X4), surgical expense (X14), and the number of HD were the most critical medical expense predictors. Furthermore, after the predictions model was built, we could use the 3 or 10 variables selected by MARS or CART, respectively, to apply SVR and XGBoost methods and achieve a better medical expense prediction model.

## 5. Conclusions

Our study developed a multiple-stage model to evaluate the one-year medical expense after discharge for those first-time CABG patients. Our model could find that the corresponding operation variables could predict one-year medical expenditure after CABG. Furthermore, postoperative complications will increase the medical expense [28]. In our results, we found that patients with kidney problems, including previous HD, PD, ESRD, renal disease, and CAD, all have a high connection with the forecast medical expenses after CABG surgery. Therefore, hospitals should enhance healthcare management on specific disease prevention, especially the CABG patients with kidney-related diseases.

Our study suggests that the SVR and XGBoost models are an adequate tool to make a medical expense prediction model, through MARS and CART feature selection. The research can bring the benefits of providing the references for medical management with specific diseases that could reduce the expense through effective control, and the government’s burdens could also be decreased.

## Figures and Tables

**Table 1:** Error measures for the performance metrics equations.

Type of Error	Metrics		Equations
Absolute error	MAE	Mean absolute error	$\frac{1}{n}\overset{n}{\underset{i=1}{\sum }}\left|\left(a^{i}-b^{i}\right)\right|$
	RMSE	Root mean square error	$\sqrt{\frac{1}{n}\overset{n}{\underset{i=1}{\sum }}{\left(a^{i}-b^{i}\right)}^{2}}$
Scaled error	MSE	Mean square error	$\frac{1}{n}\overset{n}{\underset{i=1}{\sum }}\left|{\left(a^{i}-b^{i}\right)}^{2}\right|$
	MASE	Mean absolute scaled error	$\frac{1}{n}\overset{n}{\underset{i=1}{\sum }}\frac{\left|a^{i}-b^{i}\right|}{\frac{1}{n-1}\sum_{i=2}^{n}\left|a^{i}-b^{i}\right|}$
Percentage error	MAPE	Mean absolute percentage error	$\frac{1}{n}\overset{n}{\underset{i=1}{\sum }}(|\frac{a^{i}-b^{i}}{b^{i}}|)\;\times \;100$

**Table 2:** Demographic data of new CABG patients in NHIRD from 2014 to 2017.

	Variables	Mean ± SD
Y	One-year medical expenditure after discharge (thousand NTD$)	906,693 ± 710,020
**Baseline**		
X1	Age	63.72 ± 10.65
X2	CHA2DS score	3.29 ± 1.95
X3	CCI score	4.23 ± 2.82
**Surgical variables**		
X4	Surgical expenditure(thousand NTD$)	547,037 ± 436,611
X5	Length of stay (LOS)	20.30 ± 12.02
X6	Blood transfusion, (Bag)	7.94 ± 9.29
X7	Mechanical ventilation, (Day)	4.67 ± 15.55
X8	Anastomosis vessels	2.40 ± 0.80
X9	The number of PCI vessels	1.19 ± 0.44
**One year before surgery**		
X10	Hospitalization	1.02 ± 1.31
X11	ED visits	1.27 ± 0.67
X12	Blood transfusion, (Bag)	4.08 ± 3.89
X13	Mechanical ventilation	4.74 ± 11.44
X14	Medical expenditure (thousand NTD$)	169,699 ± 247,396
X15	The number of HD Dialysis	11.96 ± 5.01
X16	The number of PD Dialysis	10.65 ± 2.91
X17	The number of PCI vessels	1.73 ± 1.13
**Comorbidities**		**N (%)**
X18	Diabetes mellitus	8142 (62.9)
X19	Hypertension	6370 (49.21)
X20	Hyperlipidemia	10,273 (79.36)
X21	Myocardial infarct	5132 (39.64)
X22	Liver cirrhosis	367 (2.84)
X23	Congestive heart failure	6687 (51.66)
X24	Coronary artery disease	12,047 (93.06)
X25	Peripheral vascular disease	2977 (23)
X26	Acute pancreatitis	432 (3.34)
X27	Malignant dysrhythmia	763 (5.89)
X28	Atrial fibrillation	1366 (10.55)
X29	Transient ischemic attack	4139 (31.97)
X30	Chronic kidney disease	3812 (29.45)
X31	Acute coronary syndrome	7384 (57.04)
X32	Chronic obstructive pulmonary disease	5036 (38.9)
X33	Stroke	4125 (31.87)
X34	Cancer	838 (6.47)
X35	Acute kidney failure	1514 (11.7)
X36	Major bleeding	3019 (23.32)
X37	Intracranial bleeding	357 (2.76)
X38	End stage renal disease	830 (6.41)
X39	Renal disease	3731 (28.82)
**Baseline**		
X40	**Gender**	
	Male	9917 (76.61)
	Female	3028 (23.39)
X41	Major illness	2108 (16.28)
**Hospital Variables**		
X42	**Hospital Area Type**	
	Central	1958 (15.13)
	Northern	8039 (62.10)
	Southern	2659 (20.54)
	Eastern	289 (2.23)
X43	**Hospital ownership**	
	Public	4558 (35.21)
	Private	8387 (64.79)
X44	**Hospital accreditation**	
	Medical center	8012 (61.89)
	Non-medcial center	4933 (38.11)

Abbreviations: CCIS: Charlson comorbidity index score; SD: standard deviation; ED: emergency department; MI: myocardial infarct; CHF: congestive heart failure; CAD: coronary artery disease; PVD: peripheral vascular disease; AF: atrial fibrillation; TIA: transient ischemic attack; CKD: chronic kidney disease; ACS: acute coronary syndrome; COPD: chronic obstructive pulmonary disease; AKF: acute kidney failure; DM: diabetes mellitus; ESRD: end-stage renal disease.

**Table 3:** Importance ranking for each predictor of medical expense, by using five different machine learning methods.

Variables		LR	SVR	CART	MARS	XGBoost	Average
X1	Age	1	0	0	0	5	1.2
X2	CHA2DS score	0	1	2	0	1	0.8
X3	CCI score	0	6	4	0	0	2
X30	Chronic kidney disease	0	7	6	0	8	4.2
X35	AKF	0	2	0	0	0	0.4
X38	ESRD	2	3	1	0	3	1.8
X39	Renal Disease	0	5	5	0	0	2
X44	Major illness	3	4	0	0	0	1.4
**Surgical variables**							
X4	Surgical expenditure	10	10	10	9	10	9.8
X6	Blood transfusion	0	0	3	0	2	1
X7	Mechanical ventilation	0	0	7	0	6	2.6
**One year before surgery**							
X12	Blood transfusion	4	0	0	0	4	1.6
X13	Mechanical ventilation	5	0	0	0	0	1
X14	Medical expenditure	8	9	9	10	9	9
X15	The number of HD Dialysis	9	8	8	8	7	8
X16	The number of PD Dialysis	6	0	0	0	0	1.2
X17	The number of PCI vessels	7	0	0	0	0	1.4

**Table 4:** Performance evaluation of prediction models after feature selection.

FS Methods	ML Method	MAE	MSE	MASE	RMSE	MAPE
LR (10 variables)	LR	0.1965	0.0813	0.3120	0.2851	0.0143
	SVR	0.1381	0.0580	0.2192	0.2407	0.0100
	MARS	0.1663	0.0591	0.2640	0.2431	0.0121
	CART	0.2024	0.0815	0.3214	0.2855	0.0148
	XGBoost	0.1458	0.0491	0.2315	0.2216	0.0106
SVR (10 variables)	LR	0.1987	0.0743	0.3155	0.2725	0.0145
	SVR	0.1345	0.0542	0.2136	0.2328	0.0097
	MARS	0.1652	0.0587	0.2623	0.2422	0.0120
	CART	0.2024	0.0815	0.3214	0.2855	0.0148
	XGBoost	0.1449	0.0491	0.2300	0.2216	0.0105
CART (10 variables)	LR	0.2002	0.0749	0.3178	0.2738	0.0146
	SVR	0.1354	0.0544	0.2149	0.2331	0.0098
	MARS	0.1652	0.0587	0.2623	0.2422	0.0120
	CART	0.2024	0.0815	0.3214	0.2855	0.0148
	XGBoost	0.1433	0.0490	0.2275	0.2214	0.0104
MARS (3variables)	LR	0.2070	0.0794	0.3287	0.2818	0.0151
	SVR	0.1302	0.0532	0.2067	0.2307	0.0094
	MARS	0.1667	0.0593	0.2647	0.2436	0.0121
	CART	0.2024	0.0815	0.3214	0.2855	0.0148
	XGBoost	0.1466	0.0499	0.2328	0.2233	0.0107
XGBoost (10 variables)	LR	0.1985	0.0739	0.3151	0.2719	0.0145
	SVR	0.1344	0.0540	0.2134	0.2324	0.0097
	MARS	0.1652	0.0586	0.2622	0.2420	0.0120
	CART	0.2024	0.0815	0.3214	0.2855	0.0148
	XGBoost	0.1443	0.0492	0.2292	0.2218	0.0105

Abbreviations: LR: linear regression; SVR: support vector regression; CART: classification and regression tree; MARS: multi-variate adaptive regression splines; AUC: area under the curve; XGBoost: extreme gradient boosting; FS: feature selection; ML: machine learning.

## Data Availability

The data presented in this study are not available on request from the corresponding author. Due to the General Data Protection Regulation, the data presented in this research are not publicly available.
